# Bovine Tuberculosis

**Published:** 1899-10

**Authors:** 


THE *Jersey Bulletin* seems very slow to learn that which has been known to the veterinary profession for many years: that an advanced case of bovine tuberculosis with marked physical signs will not respond to an ordinary injection of tuberculin, and those best acquainted with the use of this diagnostic agent always double or treble the usual amount injected. It is not a fatal charge against the merits of tuberculin, more than it would be against a failure of a vaccine point improperly prepared or used. Neither is it at all probable that so large a number of Storr's College herd contracted the disease so extensively in one short year.

